# 2-Amino-5-chloro­pyridinium salicylate

**DOI:** 10.1107/S1600536810018210

**Published:** 2010-05-22

**Authors:** Madhukar Hemamalini, Hoong-Kun Fun

**Affiliations:** a X-ray Crystallography Unit, School of Physics, Universiti Sains Malaysia, 11800 USM, Penang, Malaysia

## Abstract

In the crystal structure of the title salt, C_5_H_6_ClN_2_^+^·C_7_H_5_O_3_^−^, the protonated N atom and the 2-amino group of the cation are hydrogen bonded to the carboxyl­ate O atoms *via* a pair of N—H⋯O hydrogen bonds, forming *R*_2_^2^(8) ring motifs. These motifs are centrosymmetrically paired *via* N—H⋯O hydrogen bonds, forming a complementary donor–donor–acceptor–acceptor (*DDAA*) array. A typical intra­molecular O—H⋯O hydrogen bond is also observed in the salicylate anion. The crystal structure is further stabilized by weak C—H⋯π inter­actions.

## Related literature

For 2-amino­pyridines, see: Gellert & Hsu (1988 ▶); Banerjee & Murugavel (2004 ▶); Bis & Zaworotko (2005 ▶); Bis *et al.* (2006 ▶) and for salicylic acid, see: Cochran (1953 ▶); Singh & Vijayan (1974 ▶); Varughese & Kartha (1982 ▶). For related structures, see: Hemamalini & Fun (2010*a* ▶,*b* ▶,*c* ▶). Pourayoubi *et al.* (2007 ▶). For hydrogen-bond motifs, see: Bernstein *et al.* (1995 ▶) and for hydrogen-bonding patterns in organic salts, see: Baskar Raj *et al.* (2003 ▶). For bond-length data, see: Allen *et al.* (1987 ▶). For the stability of the temperature controller used in the data collection, see: Cosier & Glazer (1986 ▶).
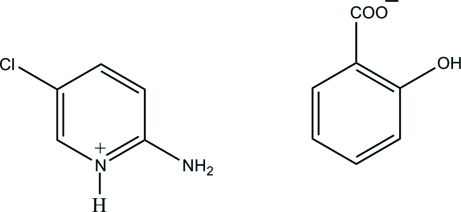


## Experimental

### 

#### Crystal data

C_5_H_6_ClN_2_^+^·C_7_H_5_O_3_^−^
*M* *_r_* = 266.68
Monoclinic, 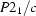

*a* = 6.7403 (6) Å
*b* = 14.5574 (12) Å
*c* = 13.2857 (9) Å
β = 115.550 (4)°
*V* = 1176.13 (16) Å^3^
*Z* = 4
Mo *K*α radiation
μ = 0.33 mm^−1^
*T* = 100 K
0.38 × 0.34 × 0.23 mm

#### Data collection

Bruker APEXII DUO CCD area-detector diffractometer
Absorption correction: multi-scan (*SADABS*; Bruker, 2009 ▶) *T*_min_ = 0.887, *T*_max_ = 0.929
19132 measured reflections
5144 independent reflections
4405 reflections with *I* > 2σ(*I*)
*R*_int_ = 0.026

#### Refinement

*R*[*F* ^2^ > 2σ(*F* ^2^)] = 0.035
*wR*(*F* ^2^) = 0.124
*S* = 1.17
5144 reflections
164 parameters
H-atom parameters constrained
Δρ_max_ = 0.66 e Å^−3^
Δρ_min_ = −0.43 e Å^−3^

### 

Data collection: *APEX2* (Bruker, 2009 ▶); cell refinement: *SAINT* (Bruker, 2009 ▶); data reduction: *SAINT*; program(s) used to solve structure: *SHELXTL* (Sheldrick, 2008 ▶); program(s) used to refine structure: *SHELXTL*; molecular graphics: *SHELXTL*; software used to prepare material for publication: *SHELXTL* and *PLATON* (Spek, 2009 ▶).

## Supplementary Material

Crystal structure: contains datablocks global, I. DOI: 10.1107/S1600536810018210/sj5004sup1.cif

Structure factors: contains datablocks I. DOI: 10.1107/S1600536810018210/sj5004Isup2.hkl

Additional supplementary materials:  crystallographic information; 3D view; checkCIF report

## Figures and Tables

**Table 1:** Hydrogen-bond geometry (Å, °) *Cg*1 is the centroid of the C7–C12 ring.

*D*—H⋯*A*	*D*—H	H⋯*A*	*D*⋯*A*	*D*—H⋯*A*
N1—H1⋯O2^i^	0.86	1.83	2.6928 (10)	178
O1—H1*B*⋯O2	0.82	1.82	2.5483 (11)	147
N2—H2*A*⋯O3^i^	0.86	1.96	2.8181 (11)	178
N2—H2*B*⋯O3^ii^	0.86	2.03	2.8321 (13)	154
C1—H1*A*⋯*Cg*1^iii^	0.93	2.57	3.3680 (11)	144

Symmetry codes: (i) 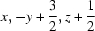; (ii) 
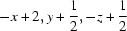
; (iii) 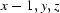.

**Fig. 1.**
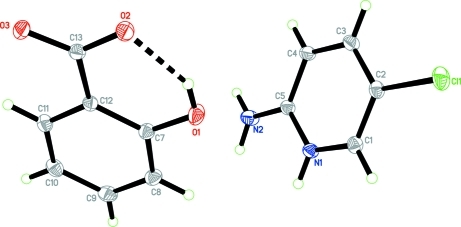
The asymmetric unit of the title compound. Displacement ellipsoids are drawn at the 50% probability level. A dashed line indicates the intramolecular hydrogen bond.

**Fig. 2.**
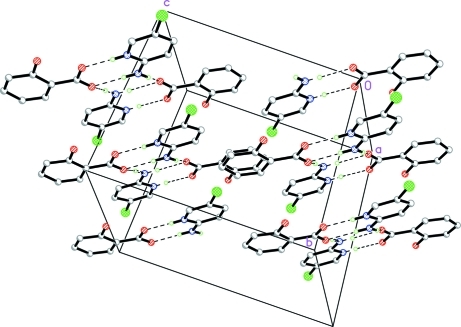
The crystal packing of the title compound, showing hydrogen-bonded (dashed lines) networks. H atoms not involved in hydrogen bond interactions are omitted for clarity.

## supplementary crystallographic information

### Comment

2-Aminopyridine is one of the most frequently used synthons in supramolecular chemistry based on hydrogen bonds (Gellert & Hsu, 1988; Banerjee & Murugavel, 2004; Bis & Zaworotko, 2005; Bis *et al.*, 2006). A series of similar complexes formed from 2-amino-5-chloropyridine and carboxylates has been reported previously (Hemamalini & Fun, 2010a,b,c). Salicylic acid (Cochran, 1953) and its derivatives are widely used as analgesic. They are also used for various gastric tympany and externally as antiseptic and antifungal agents for various skin conditions. The crystal structure of salicylic acid and its complexes, for example, antipyrine-salicylic acid (salipyrine) (Singh & Vijayan, 1974) and piperazinedione-salicylic acid (1:2) (Varughese & Kartha, 1982), have been reported in literature.  In a continuation of our studies of pyridinium derivatives, the crystal structure determination of the title compound has been undertaken.

The asymmetric unit (Fig. 1) contains one 2-amino-5-chloropyridinium cation and one salicylate anion. The proton transfer from the carboxyl group to atom N1 of 2-amino-5-chloropyridine resulted in the widening of C1—N1—C5 angle of the pyridinium ring to 122.87 (8)°, compared to the corresponding angle of 118.11 (12)° in neutral 2-amino-5-chloropyridine (Pourayoubi *et al.*, 2007). The 2-amino-5-chloropyridinium cation is essentially planar, with a maximum deviation of 0.020 (1) Å for atom Cl. The bond lengths (Allen *et al.*, 1987) and angles are normal.

In the crystal packing (Fig. 2), the protonated N1 atom and the 2-amino group (N2) are hydrogen-bonded to the carboxylate oxygen atoms (O2 and O3) via a pair of intermolecular N1—H1···O2 and N2—H2A···O3 hydrogen bonds forming an *R*^2^_2_(8)  ring motif  (Bernstein *et al.*, 1995). These motifs are centrosymmetrically paired via N—H···O hydrogen bonds, forming a complementary DDAA array (Baskar Raj *et al.* 2003). There is an intramolecular O1—H1B···O2 hydrogen bond in the salicylate anion, which generates an *S*(6) ring motif.  This motif is also observed in the crystal structure of 2-aminopyridinium salicylate (Gellert & Hsu, 1988).  The crystal structure is further stabilized by weak C—H···π  interactions (Table 1) involving the C7–C12 (centroid Cg1) ring.

### Experimental

A hot methanol solution (20 ml) of 2-amino-5-chloropyridine (64 mg, Aldrich) and salicylic acid (69 mg, Merck) were mixed and warmed over a magnetic stirrer hotplate for a few minutes. The resulting solution was allowed to cool slowly at room temperature and crystals of the title compound appeared after a few days.

### Refinement

All hydrogen atoms were positioned geometrically [C–H = 0.93 Å, N–H = 0.86 Å and O–H = 0.82 Å] and were refined using a riding model, with U_iso_(H) = 1.2 U_eq_(C, N) or 1.5 U_eq_(O).

### Crystal data

**Table:**

C_5_H_6_ClN_2_^+^·C_7_H_5_O_3_^−^	*F*(000) = 552
*M**_r_* = 266.68	*D*_x_ = 1.506 Mg m^−^^3^
Monoclinic, *P*2_1_/*c*	Mo *K*α radiation, λ = 0.71073 Å
Hall symbol: -P 2ybc	Cell parameters from 8071 reflections
*a* = 6.7403 (6) Å	θ = 2.8–35.0°
*b* = 14.5574 (12) Å	µ = 0.33 mm^−^^1^
*c* = 13.2857 (9) Å	*T* = 100 K
β = 115.550 (4)°	Block, colourless
*V* = 1176.13 (16) Å^3^	0.38 × 0.34 × 0.23 mm
*Z* = 4	

### Data collection

**Table:**

Bruker APEXII DUO CCD area-detector diffractometer	5144 independent reflections
Radiation source: fine-focus sealed tube	4405 reflections with *I* > 2σ(*I*)
graphite	*R*_int_ = 0.026
φ and ω scans	θ_max_ = 35.1°, θ_min_ = 2.2°
Absorption correction: multi-scan (*SADABS*; Bruker, 2009)	*h* = −10→9
*T*_min_ = 0.887, *T*_max_ = 0.929	*k* = −23→23
19132 measured reflections	*l* = −21→21

### Refinement

**Table:**

Refinement on *F*^2^	Primary atom site location: structure-invariant direct methods
Least-squares matrix: full	Secondary atom site location: difference Fourier map
*R*[*F*^2^ > 2σ(*F*^2^)] = 0.035	Hydrogen site location: inferred from neighbouring sites
*wR*(*F*^2^) = 0.124	H-atom parameters constrained
*S* = 1.17	*w* = 1/[σ^2^(*F*_o_^2^) + (0.0671*P*)^2^ + 0.2275*P*] where *P* = (*F*_o_^2^ + 2*F*_c_^2^)/3
5144 reflections	(Δ/σ)_max_ = 0.001
164 parameters	Δρ_max_ = 0.66 e Å^−^^3^
0 restraints	Δρ_min_ = −0.43 e Å^−^^3^

### Special details

**Table:**

Experimental. The crystal was placed in the cold stream of an Oxford Cryosystems Cobra open-flow nitrogen cryostat (Cosier & Glazer, 1986) operating at 100.0 (1) K.
Geometry. All s.u.'s (except the s.u. in the dihedral angle between two l.s. planes) are estimated using the full covariance matrix. The cell s.u.'s are taken into account individually in the estimation of s.u.'s in distances, angles and torsion angles; correlations between s.u.'s in cell parameters are only used when they are defined by crystal symmetry. An approximate (isotropic) treatment of cell s.u.'s is used for estimating s.u.'s involving l.s. planes.
Refinement. Refinement of F^2^ against ALL reflections. The weighted R-factor wR and goodness of fit S are based on F^2^, conventional R-factors R are based on F, with F set to zero for negative F^2^. The threshold expression of F^2^ > 2σ(F^2^) is used only for calculating R-factors(gt) etc. and is not relevant to the choice of reflections for refinement. R-factors based on F^2^ are statistically about twice as large as those based on F, and R- factors based on ALL data will be even larger.

### Fractional atomic coordinates and isotropic or equivalent isotropic displacement parameters (Å^2^)

**Table:**

	*x*	*y*	*z*	*U*_iso_*/*U*_eq_	
Cl1	−0.19050 (4)	0.904645 (18)	0.14133 (2)	0.02103 (8)	
N1	0.41620 (13)	0.90963 (5)	0.37992 (7)	0.01402 (14)	
H1	0.4827	0.8888	0.4469	0.017*	
N2	0.74627 (14)	0.97248 (6)	0.40024 (7)	0.01929 (16)	
H2A	0.8072	0.9514	0.4672	0.023*	
H2B	0.8229	1.0030	0.3741	0.023*	
C1	0.19743 (15)	0.89260 (6)	0.32167 (8)	0.01479 (15)	
H1A	0.1225	0.8595	0.3544	0.018*	
C2	0.08837 (15)	0.92433 (7)	0.21489 (7)	0.01471 (15)	
C3	0.20331 (15)	0.97455 (7)	0.16657 (7)	0.01588 (15)	
H3	0.1298	0.9965	0.0940	0.019*	
C4	0.42261 (15)	0.99101 (7)	0.22608 (7)	0.01550 (15)	
H4	0.4992	1.0238	0.1941	0.019*	
C5	0.53371 (15)	0.95784 (6)	0.33713 (7)	0.01379 (15)	
O1	0.56169 (12)	0.72179 (6)	0.25181 (6)	0.01955 (15)	
H1B	0.5299	0.7070	0.1871	0.029*	
O2	0.62190 (11)	0.65244 (5)	0.09104 (6)	0.01729 (14)	
O3	0.95143 (13)	0.59845 (5)	0.11872 (6)	0.01772 (14)	
C7	0.77978 (15)	0.70954 (6)	0.31440 (7)	0.01377 (15)	
C8	0.86682 (17)	0.73401 (7)	0.42726 (8)	0.01670 (16)	
H8	0.7754	0.7583	0.4567	0.020*	
C9	1.08961 (17)	0.72202 (7)	0.49541 (8)	0.01810 (17)	
H9	1.1464	0.7380	0.5704	0.022*	
C10	1.22897 (16)	0.68615 (7)	0.45190 (8)	0.01796 (17)	
H10	1.3782	0.6787	0.4975	0.022*	
C11	1.14261 (15)	0.66179 (6)	0.34034 (8)	0.01524 (15)	
H11	1.2353	0.6382	0.3113	0.018*	
C12	0.91805 (14)	0.67199 (6)	0.27039 (7)	0.01249 (14)	
C13	0.82718 (15)	0.63875 (6)	0.15205 (7)	0.01318 (15)	

### Atomic displacement parameters (Å^2^)

**Table:**

	*U*^11^	*U*^22^	*U*^33^	*U*^12^	*U*^13^	*U*^23^
Cl1	0.01164 (11)	0.02568 (13)	0.02128 (12)	−0.00140 (7)	0.00287 (8)	−0.00279 (8)
N1	0.0125 (3)	0.0153 (3)	0.0128 (3)	−0.0010 (2)	0.0041 (2)	0.0018 (2)
N2	0.0127 (3)	0.0256 (4)	0.0160 (3)	−0.0046 (3)	0.0029 (3)	0.0045 (3)
C1	0.0129 (3)	0.0144 (4)	0.0164 (4)	−0.0011 (3)	0.0057 (3)	−0.0001 (3)
C2	0.0116 (3)	0.0160 (4)	0.0147 (3)	−0.0002 (3)	0.0039 (3)	−0.0016 (3)
C3	0.0148 (4)	0.0178 (4)	0.0127 (3)	0.0013 (3)	0.0038 (3)	0.0009 (3)
C4	0.0150 (4)	0.0173 (4)	0.0136 (3)	−0.0003 (3)	0.0056 (3)	0.0025 (3)
C5	0.0122 (3)	0.0149 (4)	0.0131 (3)	−0.0006 (3)	0.0044 (3)	0.0007 (3)
O1	0.0123 (3)	0.0292 (4)	0.0164 (3)	0.0026 (2)	0.0054 (2)	−0.0033 (3)
O2	0.0128 (3)	0.0237 (3)	0.0128 (3)	0.0018 (2)	0.0031 (2)	−0.0024 (2)
O3	0.0176 (3)	0.0215 (3)	0.0145 (3)	0.0046 (2)	0.0074 (3)	−0.0015 (2)
C7	0.0133 (3)	0.0146 (3)	0.0134 (3)	−0.0001 (3)	0.0057 (3)	−0.0004 (3)
C8	0.0184 (4)	0.0189 (4)	0.0139 (3)	0.0001 (3)	0.0079 (3)	−0.0016 (3)
C9	0.0209 (4)	0.0187 (4)	0.0117 (3)	−0.0012 (3)	0.0042 (3)	−0.0008 (3)
C10	0.0158 (4)	0.0181 (4)	0.0150 (4)	0.0010 (3)	0.0019 (3)	0.0000 (3)
C11	0.0133 (3)	0.0153 (4)	0.0155 (3)	0.0012 (3)	0.0047 (3)	−0.0005 (3)
C12	0.0123 (3)	0.0130 (3)	0.0116 (3)	0.0004 (3)	0.0047 (3)	−0.0001 (3)
C13	0.0138 (3)	0.0135 (3)	0.0118 (3)	0.0004 (3)	0.0052 (3)	0.0004 (3)

### Geometric parameters (Å, °)

**Table:**

Cl1—C2	1.7280 (9)	O1—H1B	0.8200
N1—C5	1.3534 (12)	O2—C13	1.2821 (11)
N1—C1	1.3603 (12)	O3—C13	1.2496 (11)
N1—H1	0.8600	C7—C8	1.4002 (13)
N2—C5	1.3285 (12)	C7—C12	1.4069 (12)
N2—H2A	0.8600	C8—C9	1.3901 (14)
N2—H2B	0.8600	C8—H8	0.9300
C1—C2	1.3662 (13)	C9—C10	1.3992 (14)
C1—H1A	0.9300	C9—H9	0.9300
C2—C3	1.4057 (13)	C10—C11	1.3846 (13)
C3—C4	1.3635 (13)	C10—H10	0.9300
C3—H3	0.9300	C11—C12	1.4012 (13)
C4—C5	1.4204 (12)	C11—H11	0.9300
C4—H4	0.9300	C12—C13	1.5001 (12)
O1—C7	1.3527 (11)		
			
C5—N1—C1	122.87 (8)	O1—C7—C8	117.84 (8)
C5—N1—H1	118.6	O1—C7—C12	122.33 (8)
C1—N1—H1	118.6	C8—C7—C12	119.81 (8)
C5—N2—H2A	120.0	C9—C8—C7	120.10 (8)
C5—N2—H2B	120.0	C9—C8—H8	119.9
H2A—N2—H2B	120.0	C7—C8—H8	119.9
N1—C1—C2	119.66 (8)	C8—C9—C10	120.45 (8)
N1—C1—H1A	120.2	C8—C9—H9	119.8
C2—C1—H1A	120.2	C10—C9—H9	119.8
C1—C2—C3	119.63 (8)	C11—C10—C9	119.39 (9)
C1—C2—Cl1	119.87 (7)	C11—C10—H10	120.3
C3—C2—Cl1	120.48 (7)	C9—C10—H10	120.3
C4—C3—C2	119.98 (8)	C10—C11—C12	121.20 (8)
C4—C3—H3	120.0	C10—C11—H11	119.4
C2—C3—H3	120.0	C12—C11—H11	119.4
C3—C4—C5	119.78 (8)	C11—C12—C7	119.03 (8)
C3—C4—H4	120.1	C11—C12—C13	119.80 (8)
C5—C4—H4	120.1	C7—C12—C13	121.11 (8)
N2—C5—N1	119.03 (8)	O3—C13—O2	123.67 (8)
N2—C5—C4	122.89 (8)	O3—C13—C12	119.32 (8)
N1—C5—C4	118.08 (8)	O2—C13—C12	117.00 (7)
C7—O1—H1B	109.5		
			
C5—N1—C1—C2	0.46 (14)	C8—C9—C10—C11	−0.61 (15)
N1—C1—C2—C3	−0.25 (14)	C9—C10—C11—C12	−0.31 (15)
N1—C1—C2—Cl1	−178.88 (7)	C10—C11—C12—C7	1.35 (14)
C1—C2—C3—C4	0.27 (14)	C10—C11—C12—C13	−175.67 (9)
Cl1—C2—C3—C4	178.89 (7)	O1—C7—C12—C11	179.72 (8)
C2—C3—C4—C5	−0.47 (14)	C8—C7—C12—C11	−1.48 (13)
C1—N1—C5—N2	179.07 (9)	O1—C7—C12—C13	−3.30 (14)
C1—N1—C5—C4	−0.64 (13)	C8—C7—C12—C13	175.51 (8)
C3—C4—C5—N2	−179.05 (9)	C11—C12—C13—O3	3.25 (13)
C3—C4—C5—N1	0.64 (13)	C7—C12—C13—O3	−173.71 (8)
O1—C7—C8—C9	179.44 (9)	C11—C12—C13—O2	−177.95 (8)
C12—C7—C8—C9	0.58 (14)	C7—C12—C13—O2	5.09 (13)
C7—C8—C9—C10	0.47 (15)		

### Hydrogen-bond geometry (Å, °)

**Table:**

Cg1 is the centroid of the C7–C12 ring.

**Table:**

*D*—H···*A*	*D*—H	H···*A*	*D*···*A*	*D*—H···*A*
N1—H1···O2^i^	0.86	1.83	2.6928 (10)	178
O1—H1B···O2	0.82	1.82	2.5483 (11)	147
N2—H2A···O3^i^	0.86	1.96	2.8181 (11)	178
N2—H2B···O3^ii^	0.86	2.03	2.8321 (13)	154
C1—H1A···Cg1^iii^	0.93	2.57	3.3680 (11)	144

Symmetry codes: (i) *x*, −*y*+3/2, *z*+1/2; (ii) −*x*+2, *y*+1/2, −*z*+1/2; (iii) *x*−1, *y*, *z*.
